# A case report of mediastinal cavernous hemangioma

**DOI:** 10.1002/ccr3.4698

**Published:** 2021-08-25

**Authors:** Davoud Ouladdameshghi, Mohsen Eshraghi, Alireza Shahhamzeh, Mohammad Mehdi Riyah, Maedeh Alsadat Fatemi, Danial Fazilat‐Panah, Mansoureh Dehghani

**Affiliations:** ^1^ Clinical Research Development Qom Medical University Qom Iran; ^2^ Department of Surgery School of Medicine Qom University of Medical Sciences Qom Iran; ^3^ School of Medicine Shahid Beheshti Hospital Qom University of Medical Sciences Qom Iran; ^4^ Qom University of Medical Sciences Qom Iran; ^5^ Cancer Research Center Babol University of Medical Sciences Babol Iran; ^6^ Cancer Research Centre Neyshabur University of Medical Sciences Neyshabur Iran

**Keywords:** case report, cavernous, mediastinal hemangioma

## Abstract

When faced with a hypervascular mediastinal tumor, mediastinal hemangioma should be taken into consideration. Although it is uncommon, considering this important diagnosis may avoid a possible extensive surgery that is not necessary.

## Graphical abstract

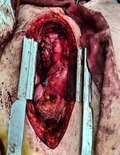

## 1 INTRODUCTION

Mediastinal hemangioma is an uncommon benign tumor. Here, we report a 36‐year‐old woman presenting with dyspnea who underwent surgery to excise a mediastinal mass. The final diagnosis was cavernous hemangioma. Preoperative and intraoperative consideration of mediastinal hemangioma is essential in order to avoid unnecessary extensive surgery.

Mediastinal hemangioma is a rare tumor that consists of only 0.5% of masses located in the mediastinum. It has histological varieties, which are classified into cavernous, capillary, and venous types.^1^

This tumor is generally well circumscribed, but may be infiltrated into the surrounding tissue. It generally requires surgical excision, despite its benign nature. Mediastinal hemangiomas have no specific symptoms. A variety of clinical manifestations might occur, such as a cough, mild dyspnea, respiratory distress, or chest pain.^2^, ^3^

Here, we report a case of mediastinal hemangioma presenting with dyspnea, in the time of COVID‐19 pandemia.

## 2 CASE PRESENTATION

**FIGURE 1:**
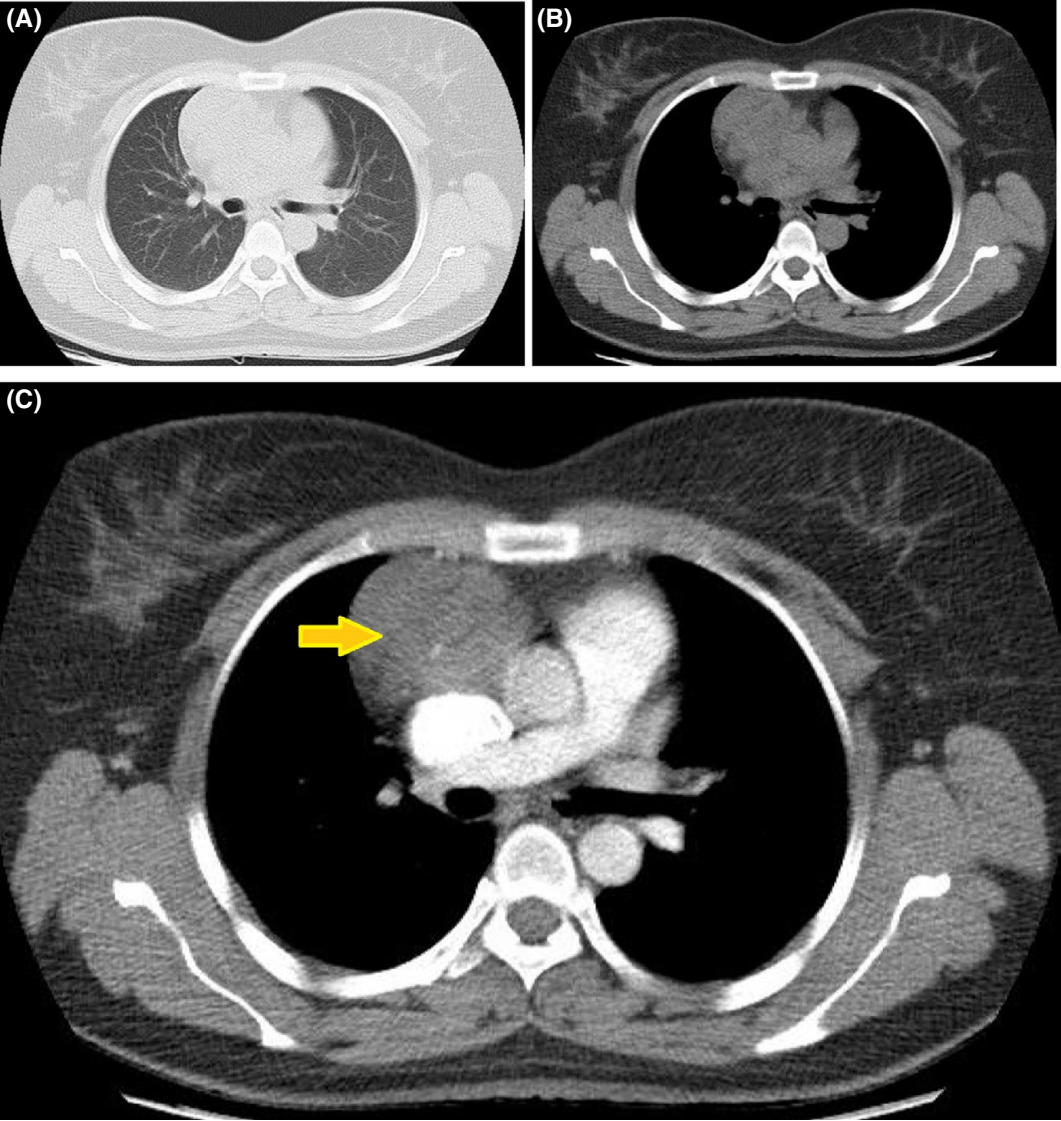
Axial CT scan demonstrating a heterogeneous lesion in the right anterior mediastinum (yellow arrow). (A): Lung window, (B): mediastinal window, and (C): after contrast administration, arterial phase

**FIGURE 2:**
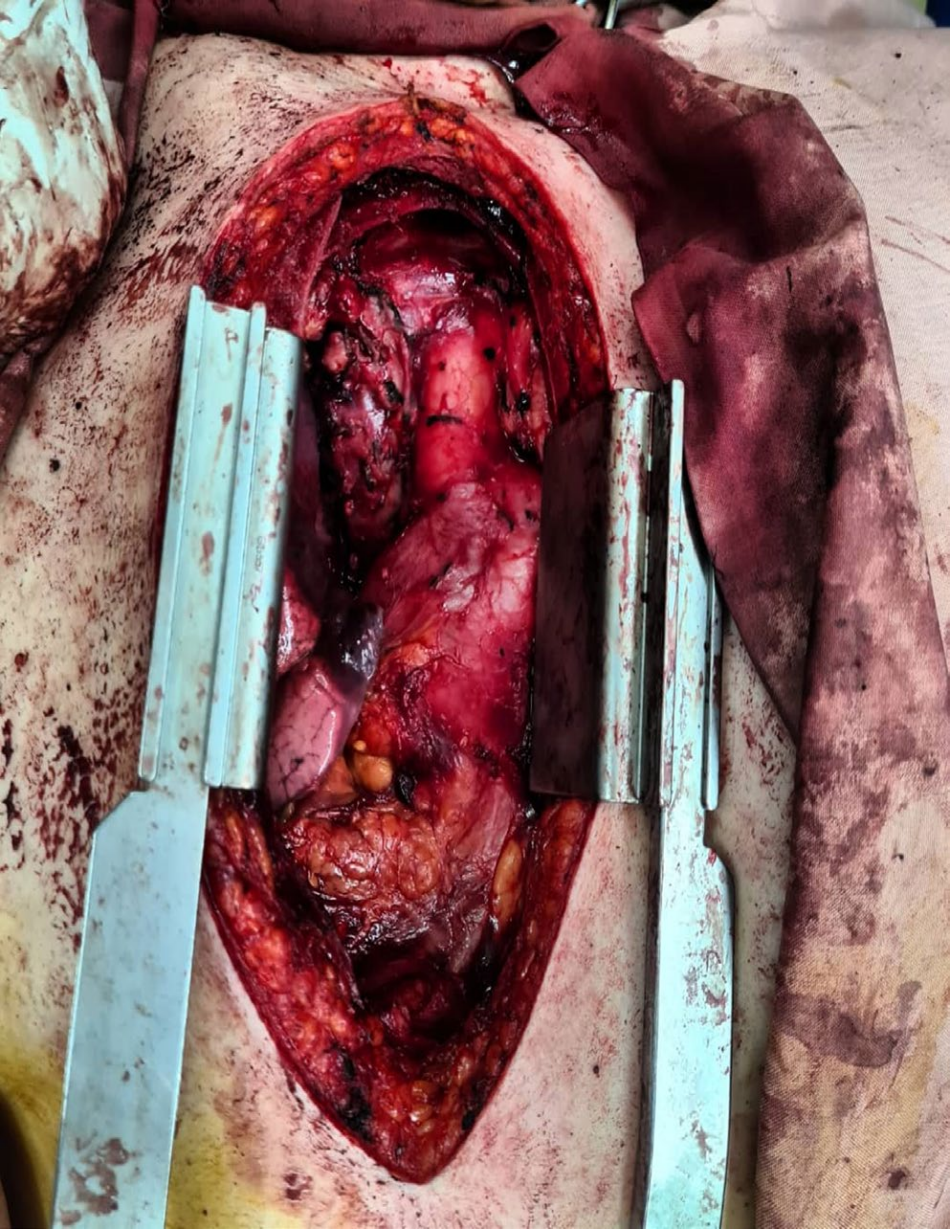
Surgical view of tumor. After median sternotomy was performed, a hypervascular soft tissue mass in the anterior mediastinal region, measuring 10 × 10 × 6 cm, was exposed, which was completely resected successfully

**FIGURE 3:**
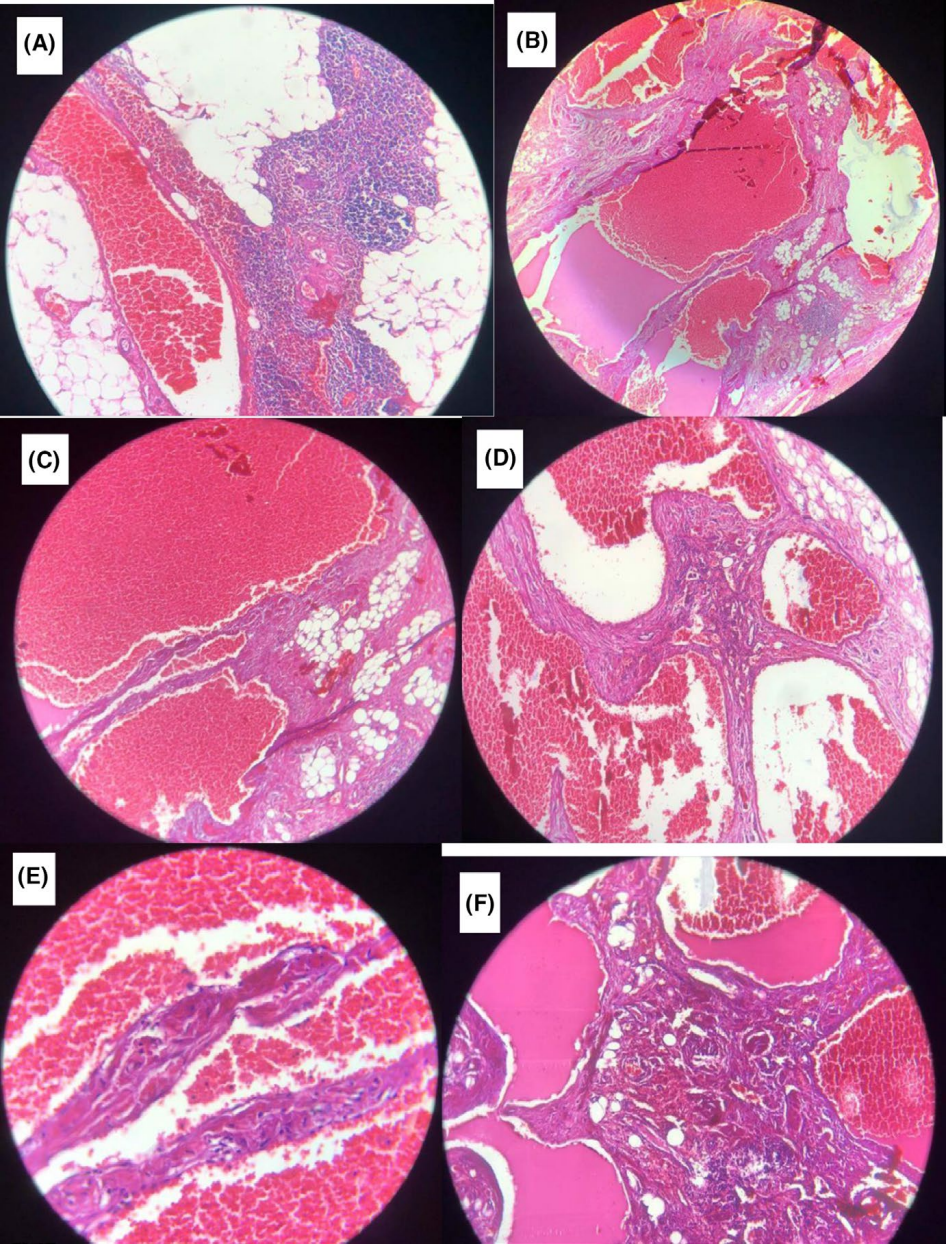
Microscopic pathological findings. The lesion is composed of dilated vascular sinusoids, lined by endothelium containing blood and proteinaceous material

A healthy 36‐year‐old woman with no past medical and familia histories presented to the clinic with a chief complaint of dyspnea since three months ago. The dyspnea was persistent during the time without limiting the daily activities. Also, there were no aggravating and alleviating factors. Vital signs at the presentation were stable. The patient had normal breath sounds in all segments of the lungs. Initial paraclinical evaluations including complete blood tests, blood biochemistry assessment, and COVID‐19 PCR test were all normal. Chest radiograph showed a mediastinal widening, indicating the presence of a mediastinal mass. Therefore, a CT scan was performed that demonstrated a heterogeneous lesion in the right anterior mediastinum (Figure 1). The lesion measured 84 × 47 mm, which was reported to be suggestive of thymoma or teratoma, and biopsy or excision was recommended. Other CT scan findings consisted of a 5 mm pulmonary nodule at the lateral segment of the left lower lobe, fibrotic bands at lower lobes, and splenomegaly. There was no opacity, volume change, pleural effusion, or chest wall abnormality. An invasive thymic tumor or soft tissue tumor with high vasculature was suspected mainly because of its location and a radiological extent, biopsy was not planned due to high risk of major bleeding, and tumor excision through median sternotomy was performed by an attending thoracic surgeon at our institute. Because a complete resection was feasible, no intraoperative frozen section examination was executed (Figure 2). The postoperative course did not have a mentionable event, and the patient was discharged on the fourth postoperative day. According to the pathology report, the macroscopic findings of the specimen were one piece of light‐tan tissue measuring 10 × 10 × 6 cm, containing solid cystic parts with hematoma inside. Microscopic examination of 12 pieces in 12 blocks demonstrated thymic tissue with encapsulated neoplasm consisting variable sized and dilated vascular sinusoids, lined by endothelium containing blood and pertinacious material (Figure 3). The conclusive pathological diagnosis was cavernous hemangioma of mediastinum.

## 3 DISCUSSION

Mediastinal masses are divided into anterior, middle, and posterior according to their location. Each of these subclasses is suggestive of a different differential diagnosis, but a mass in any of the three locations can demonstrate hypervascular features.^4^

Mediastinal hemangioma is a rare tumor, which was first reported by Shenan in 1914. These tumors are classified as capillary, cavernous, or venous, according to the size of the vascular structure. More than 90 percent of them are cavernous or capillary.^5^ In 1987, Cohen et al published a detailed analysis of 103 cases.^6^ In 2006, Yamazaki et al reported a case similar to the case in the present study (cavernous hemangioma of anterior mediastinum) and did a literature survey. They found a total of 61 cases of mediastinal hemangioma reported in Japan over 50 years. This study also declared that a total of fewer than 110 cases have been reported overall.^7^

The International Society for the Study of Vascular Anomalies (ISSVA) classification has classified mediastinal hemangioma into benign vascular tumors. These tumors, which mostly present before the age of 35, may occur at the anterior or posterior mediastinum, although the anterior location is slightly more prevalent. There are also reports of middle mediastinal location. Differential diagnoses of hypervascular anterior mediastinal masses, which should be considered, include castleman disease, paraganglioma, vascular malformation, hypervascular metastasis, and ectopic parathyroid adenoma.^1^, ^7^, ^8^

As many as 50% of the reported cases of mediastinal hemangioma have been asymptomatic, discovered during a routine checkup imaging or an investigation for some other reason. Spontaneous regression is also possible similar to angiomas in other sites. However, many display clinical features of proliferation depending on the location and the underlying condition. Depending on the origin and size, intrathoracic lesions may be associated with cough, dyspnea, chest pain, hemoptysis, congestive cardiac failure, respiratory distress, or a clinical picture referred to as the Kasabach‐Merritt syndrome, which is a result of platelet consumption inside the angioma.^8^, ^9^ The patient introduced in this report presented with a history of dyspnea without a known underlying condition, which was an indicator for COVID‐19 evaluation and testing as the first step. Although the history suggested a relatively longer time period unlike in COVID‐19 infection, it was mandatory to rule out this diagnosis in the pandemic era especially with its impact on the mortality of patients with malignancies.^10^, ^11^

Although most cases are generally envisioned as well‐circumscribed masses on imaging studies, some can display a wide range of radiological features like dumbbell tumors or infiltrative lesions.^12^

Thoracic CT scans generally demonstrate a heterogeneous well‐circumscribed mass, but there are few reports of infiltrative appearance. Multiple, punctate, and rounded calcifications are reported in many cases. McAdams et al performed a retrospective study of CT scan features in 14 cases of mediastinal hemangioma. Ten demonstrated heterogeneous attenuation after contrast administration, 6 cases had a pattern of central increased attenuation similar to or greater than that of the vascular structures, and 2 cases showed a mixed pattern of increased central and peripheral attenuation. In one case, only peripheral attenuation was reported.^13^

Early reports indicate that there is little diagnostic use in transthoracic needle aspiration biopsy of mediastinal hemangiomas.^14^ It is recommended to surgically exercise the two more in order to provide both diagnoses and treatment. Unlike other mediastinal tumors, even a subtotal resection would be feasible for hemangiomas without increased risk of local recurrence, hemorrhagic morbidity, malignant transformation, or becoming symptomatic.^6^ Therefore, hemangioma should be raised as a differential diagnosis investigated in mediastinal tumors to avoid extensive surgery. Yobita et al recently reported an extensive surgical resection of an infiltrative mediastinal mass, with the part of the lower lobe of left lung and diaphragm, which was diagnosed as hemangioma after pathologic examination. The resected lung and diaphragm turned out to have no histological invasion, and the adhesion only consisted of fibrous tissue.^12^

In addition to surgery, utilization of other treatments including radiotherapy or the support of veno‐venous extracorporeal membrane oxygenation (VV‐ECMO) is reported, especially in critical situations such as massive hemoptysis and Kasabach‐Merritt syndrome.^9^, ^15^ Modern radiotherapy techniques have special abilities to delineate the tumor and spare the organ at risks effectively, which may help to improve tumor coverage and decrease the dose to the normal tissues.^16^, ^17^ Besides, there are promising new data on the underlying molecular mechanisms of different cancer, which may affect the treatment of these benign tumors with the least side effects.^18^, ^19^, ^20^, ^21^

## 4 CONCLUSION

When faced with a hypervascular mediastinal tumor, mediastinal hemangioma should be taken into consideration. Although it is uncommon, considering this important diagnosis may avoid a possible extensive surgery that is not necessary.

## CONFLICT OF INTEREST

The authors declare no conflict of interest. The study was fully funded by the Deputy of Research and Technology of Mashhad University of Medical Sciences.

## AUTHOR CONTRIBUTIONS

D.O, M.E., and A. Sh. contributed to conception, design, and drafting of the manuscript. M.M.R. and M.D. contributed to data collection. M.A.F. and D.F. contributed to drafting of the manuscript. D.F. supervised the study. All authors approved the final version for submission.

## ETHICAL APPROVAL

The study was approved by Qom University of Medical Sciences. The study conforms to recognize the standards of Declaration of Helsinki. Written informed consent was obtained from the patient.

## Data Availability

The data sets used and/or analyzed during the current study are available from the corresponding authors per request.
